# DaxibotulinumtoxinA for Injection in Adults with Cervical Dystonia: Clinical Insights from a Real‐World Early Experience Program

**DOI:** 10.1002/mdc3.70477

**Published:** 2026-02-03

**Authors:** Laxman Bahroo, Aaron Ellenbogen, Robert A. Hauser, Han S. Lee, Peter McAllister, Atul T. Patel, Rashid Kazerooni, Todd M. Gross, Julia Sparrow, Conor J. Gallagher, David A. Hollander

**Affiliations:** ^1^ MedStar Georgetown University Hospital Washington Washington DC USA; ^2^ Quest Research Institute Farmington Hills Michigan USA; ^3^ University of South Florida Tampa Florida USA; ^4^ The Permanente Medical Group San Leandro California USA; ^5^ New England Institute for Neurology and Headache Stamford Connecticut USA; ^6^ Kansas City Bone & Joint Clinic Overland Park Kansas USA; ^7^ Revance Nashville Tennessee USA

**Keywords:** cervical dystonia, MeSH: botulinum toxins, Type A, movement disorders, Non‐MeSH: DaxibotulinumtoxinA, spasmodic torticollis

## Abstract

**Background:**

DaxibotulinumtoxinA for injection (DAXI), a novel botulinum toxin (BoNT) formulated with a custom‐engineered peptide, was recently approved for treating cervical dystonia (CD). DAXI demonstrated a long duration of symptom relief in Phase 3 trials.

**Objective:**

To report findings from PrevU, an early experience, real‐world observational program initiated following DAXI's FDA approval.

**Methods:**

Movement disorder specialists were provided with DAXI to treat CD patients per routine clinical practice, and were surveyed on dosing patterns and treatment intervals across the first three cycles of DAXI as doses were optimized.

**Results:**

A total of 234 CD patients (206 receiving BoNT treatment and 28 BoNT‐naïve) received a total of 589 DAXI treatments. Among patients with prior BoNT therapy, breakthrough symptoms within 12 weeks of prior BoNT therapy was the most common reason for patient selection (76.2%), and onabotulinumtoxinA was the most common prior toxin (68.4%). DAXI dose was titrated from a mean of 244.4 U (median 250 U) to 314.7 U (median 300 U) by Cycle 3. The ratio of DAXI dose to normalized prior BoNT dose was approximately 1.1:1 at initiation and 1.4:1 at Cycle 3. The mean (SD) time to re‐treatment across all DAXI cycles was 16.1 (5.6) weeks for all patients and 15.7 (4.5) weeks for patients with a history of breakthrough before 12 weeks with prior toxin. The most common adverse events were muscle weakness (2.4% of treatments), injection pain (1.4% of treatments), and dysphagia (0.3% of treatments).

**Conclusions:**

In this real‐world setting, DAXI demonstrated extended clinical benefit and a favorable safety profile.

Repeat injections of botulinum toxin (BoNT) is the well‐established first‐line treatment for cervical dystonia (CD),^1^, ^2^ a neurological condition in which patients experience involuntary movements and posturing of the head, neck, and shoulders. Head tremor and pain are common in CD and, together with the dystonia and abnormal posturing, impair the patient's quality of life.^3^, ^4^, ^5^, ^6^, ^7^ Current product labels and insurance reimbursement practice limit re‐injection frequency to no sooner than 12 weeks.^8^, ^9^, ^10^, ^11^ As a result, patients frequently experience inadequate symptom relief before their next treatment, with many requesting shorter treatment intervals or longer‐lasting treatments. The mismatch between duration and re‐treatment interval with conventional BoNTs can result in a repeated pattern of decreasing then increasing symptom relief, often referred to as a “rollercoaster” or “yo‐yo” effect. Therefore, durability of response continues to be an unmet need for CD.^12^, ^13^

DaxibotulinumtoxinA for injection (DAXI; DAXXIFY®, Revance Therapeutics, Inc., Nashville, TN, USA) is a novel type A BoNT approved for the treatment of CD by the US Food and Drug Administration (FDA) in August 2023.^14^ DAXI is formulated with a custom‐engineered peptide^14^ known as RTP004. Studies have demonstrated that the positively charged RTP004 facilitates greater neuronal cell binding of neurotoxin, which may increase neuronal bioavailability and reduce nonspecific diffusion to adjacent tissues.^15^, ^16^, ^17^, ^18^ As such, a lower amount of core neurotoxin may be necessary to achieve a desired effect relative to conventional BoNTs. Phase 3 trials of DAXI for CD (ASPEN‐1 and ASPEN‐OLS)^19^, ^20^ demonstrated a median of 20–24 weeks for patients to return to pre‐treatment status and a median of 16–17 weeks to request and receive re‐treatment, with approximately half of peak efficacy remaining.^21^ This extended duration of symptom relief was coupled with lower reported rates of dysphagia and muscle weakness (both <5%), compared with the rates previously reported for conventional BoNTs.^19^, ^20^

While pivotal trials are designed to evaluate the safety and efficacy of new pharmaceutical agents, the design and inclusion criteria of these studies may not fully reflect how medications will be utilized in clinical practice. The current early experience program was intended to provide a better understanding of how physicians select CD patients, administer DAXI, and perform re‐treatments in a real‐world clinical setting, outside the confines of a clinical study.

## Methods

### Study Design

This was a retrospective, observational summary of physician experience, based on treatment of patients with CD in an early experience program (PrevU), initiated following FDA approval of DAXI for the treatment of CD. The PrevU program was conducted across 16 practices in the US from August 30, 2023 through December 31, 2024. As all data were obtained as part of patients’ routine medical care, informed patient consent was not necessary for this work.

### Patients and Treatment

Adult patients with CD were selected and treated in accordance with routine clinical practice by their treating physicians; patient selection and treatment decisions were not governed by a study protocol. Both BoNT‐naïve patients and patients with a history of BoNT treatment for CD were included. All physicians were movement disorder specialists with extensive experience using BoNTs in the treatment of CD. Physicians were given sufficient samples of DAXI to treat their selected patients for 3 cycles.

### Outcome Measures and Analyses

Physicians summarized the reason for selecting individual patients for treatment with DAXI, as well as prior BoNT type and dose, when applicable. The reasons for selection were grouped into the following categories: experienced breakthrough symptoms before 12 weeks on a previous BoNT, tolerability issues with current BoNT therapy, satisfied with a previous BoNT, nonclinical obstacles to treatment, or BoNT‐naïve. More than one characteristic could be noted for each patient.

Practitioners provided information on the doses administered, the reasons for re‐treatment, and re‐treatment intervals for up to the first three cycles of treatment with DAXI, which covered the period during which treatment parameters were being optimized. Time to re‐treatment was calculated as weeks between successive treatments. Patients who received a BoNT other than DAXI at treatment Cycles 2 or 3 were considered to have discontinued DAXI at the end of the preceding cycle. DAXI samples were only provided for the first three DAXI treatments. To broadly approximate the relationship between prior BoNT dose and DAXI dose, for the purposes of this analysis, it was assumed that onabotulinumtoxinA and incobotulinumtoxinA units were comparable, abobotulinumtoxinA units were divided by three, and rimabotulinumtoxinB units were divided by 50.

Descriptive statistical analyses were performed using SAS 9.4 (SAS Institute Inc., Cary, NC). Descriptive statistics included frequency and percent for categorical variables, count, mean, standard deviation (SD), median, minimum (min), and maximum (max) for quantitative variables. The primary analysis was performed on observed cases within each cycle. Due to variability in the date of initial treatment and the length of re‐treatment intervals for individual patients, the number of patients with data in each treatment cycle varied at completion of the experience program. In order to evaluate potential bias from variance in the number of patients in each cycle, the analyses were repeated in the constant cohort of patients who received all three treatments.

## Results

### Analysis Population

In this early experience program, 16 physicians treated 234 CD patients with DAXI for up to three treatment cycles. A total of 589 DAXI treatments were administered. Of the treated patients, 206 (88.0%) had previously been treated with a BoNT for CD and 28 (12.0%) were BoNT treatment naïve (Table 1). The most common prior treatment was onabotulinumtoxinA, accounting for 68.4% (141 of 206) of the treatment‐experienced patients, followed by incobotulinumtoxinA (14.6%, 30 of 206), abobotulinumtoxinA (5.8%, 12 of 206), and rimabotulinumtoxinB (3.4%, 7 of 206). Ten patients had previously participated in the Phase 3 clinical trials of DAXI, which ended in May of 2021. These patients returned to a different BoNT in the interim.

**TABLE 1:** Treatment history

Category	*N* (%)
All treated patients	234
Treatment‐naïve	28 (12.0)
Treatment‐experienced	206 (88.0)
Experienced breakthrough symptoms within 12 weeks of prior BoNT	157 (76.2)
Satisfied with prior BoNT	31 (15.0)
Experienced nonclinical obstacles with prior BoNT (eg, long‐distance travel)	29 (14.1)
Experienced tolerability‐limited dosing with prior BoNT	11 (5.3)

*Note*: Multiple categories were selected per patient; percentages sum to >100%.

Abbreviation: BoNT, botulinum toxin.

A majority (76.2%, 157 of 206) of the BoNT treatment‐experienced patients were described by their treating physicians as having experienced breakthrough symptoms within 12 weeks of receiving their prior BoNT therapy. Only 15.0% (31 of 206) of CD patients were considered by their physicians as being satisfied with their previous BoNT therapy.

**Figure 1:**
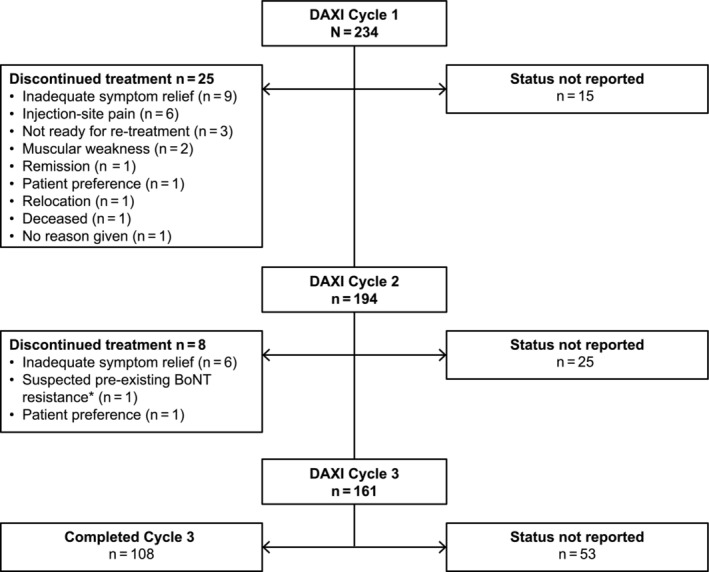
Patient disposition in the PrevU Experience Program. After each cycle patients were followed until subsequent treatment. Patients with “status not reported” were either maintaining efficacy at time of data cut‐off or lost‐to‐follow‐up. *The patient had no history of prior BoNT treatment, showed inadequate response to DAXI 150 U dose at Cycle 1 and 200 U dose at Cycle 2, and failed a frontalis antibody test (FTAT) 6 weeks after the second DAXI treatment. BoNT, botulinum toxin; DAXI, daxibotulinumtoxinA.

Of the 234 patients treated, a total of 194 patients received at least two cycles of DAXI and 161 patients received three cycles (Fig. 1). Twenty‐five patients (11%) discontinued DAXI treatment following Cycle 1, and 8 patients (3%) discontinued following Cycle 2. The most common reasons for discontinuation were inadequate symptom relief during dose titration (15, 6.4%), injection site pain (6, 2.6%), not ready for re‐treatment (3, 1.3%), muscular weakness (2, 0.9%), and remission of symptoms (1, 0.4%). One BoNT‐naïve patient discontinued after Cycle 2 due to an inadequate primary efficacy response following DAXI treatments of 150 U and 200 U, followed by a negative response to a frontalis antibody test (FTAT), suggesting a preexisting resistance to BoNT Type A.

### Dosing Characteristics

**Figure 2:**
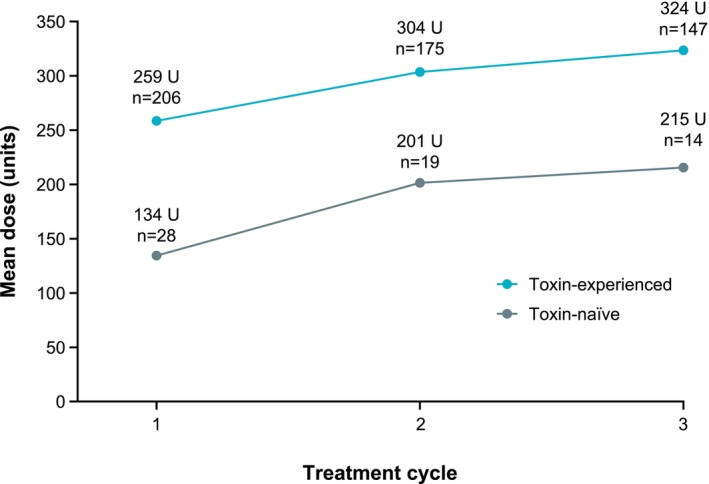
DAXI dose and titration across 3 treatment cycles in toxin‐naïve and toxin‐experienced patients. U, units.

The DAXI doses administered across the three treatment cycles are summarized in Table 2. The mean starting dose was 244.4 U (median 250 U) and by Cycle 3 was titrated to a mean of 314.7 U (median 300 U). Dose increases were generally greater at Cycle 2 (mean: 46.1 U) and smaller at Cycle 3 (mean: 23.4 U). BoNT‐naïve patients were generally started at a lower dose than were BoNT‐experienced patients, with a mean of 134 U and 259 U, respectively. This pattern of lower dosing for BoNT‐naïve patients was observed at all three treatment cycles (see Fig. 2). In the treatment‐experienced group, the ratio of DAXI dose to prior BoNT started at 1.1:1 and increased to 1.4:1 by Cycle 3 (Table 2). Across all treatment cycles, the maximum administered DAXI dose was 800 U for BoNT‐experienced patients, and 400 U for BoNT‐naïve patients.

**TABLE 2:** *DAXI* dosing patterns over 3 treatment cycles

	Cycle 1 (*n* = 234)	Cycle 2 (*n* = 194)	Cycle 3 (*n* = 161)
Dose, units–mean (SD)	244.4 (105.6)	293.9 (122.8)	314.7 (131.7)
Median (min–max)	250 (25–600)	300 (35–750)	300 (60–800)
Dose change, units–mean (SD)	–	46.1 (62.9)	23.4 (49.5)
Ratio–mean (SD)^a^	1.14 (0.45)	1.30 (0.54)	1.38 (0.56)
Median (min–max)^a^	1.00 (0.44–3.80)	1.15 (0.33–4.30)	1.25 (0.45–3.75)

Abbreviations: BoNT, botulinum toxin; DAXI, daxibotulinumtoxinA; SD, standard deviation.

^a^ Ratio of DAXI to prior BoNT in onabotulinumtoxinA equivalents.

### Re‐Treatment Intervals with DAXI

**Figure 3:**
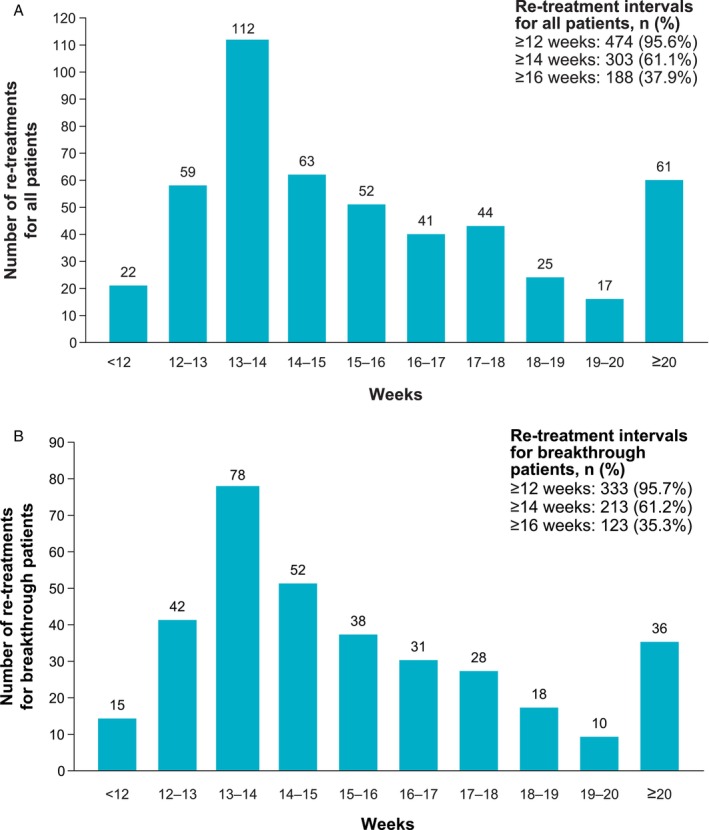
Time to re‐treatment with DAXI among (A) all patients and (B) patients with breakthrough symptoms earlier than 12 weeks on prior BoNT therapy. Time intervals (weeks) represent the duration from the initial week inclusive to the last week exclusive (eg, 12–13 represents ≥12 − <13).

The physicians re‐treated patients with DAXI at a mean (SD) interval of 16.1 (5.6) weeks across all cycles. Mean weeks to re‐treatment was similar across all treatment cycles (16.7, 15.7, and 15.5 for Cycles 1, 2, and 3, respectively). A large proportion of treatment intervals (61.1%) were 14 weeks or greater and more than one‐third (37.9%) were 16 weeks or greater (see Fig. 3). The mean (SD) re‐treatment interval was 15.7 (4.5) weeks for patients with breakthrough symptoms within 12 weeks with prior therapy and 17.5 (9.0) for BoNT‐naïve patients.

### Reason for Re‐Treatment

At the time of re‐treatment, physicians documented whether they were treating to “maintain symptom relief” or “address a decline in symptom relief.” Based on this physician feedback, close to half (50.8%) of all treatments were administered with to avoid re‐emergence of symptoms (Table 3). Dose titration between cycles was typically greater (mean 46 U vs 14 U) for those patients treated for a “decline in symptom relief” compared with those treated to “maintain symptom relief,” respectively.

**TABLE 3:** Reason for re‐treatment and DAXI dose titration across all cycles

	*n* (%)^a^	Mean	Median	Increased	No change	Decreased
Address a decline in symptom relief	215 (49.2)	46 U	50 U	63%	29%	8%
Maintain symptom relief	222 (50.8)	14 U	0 U	32%	57%	11%

^a^ From 437 treatments with documented reason for re‐treatment.

### Cohort Receiving all 3 Treatments

Results for the subset of patients receiving three treatments (*n* = 161) were similar to those analyzed for all patients. Across Cycles 1, 2, and 3, the mean dose was 248 U, 291 U, and 315 U, respectively, the mean dose ratio was 1.11:1, 1.28:1, and 1.38:1, the mean dose increase was 43 U and 23 U (for Cycles 2 and 3, respectively), and the mean re‐treatment interval was 15.8, 15.8, and 15.5 weeks, respectively.

### Adverse Events

Across the three treatment cycles, a total of 26 adverse events (4.4% of 589 treatments) were reported (Table 4). The most frequently reported adverse events were muscle weakness (2.4%), injection pain (1.4%), and dysphagia (0.3%). In addition, one case of neck pain and one case of upper respiratory infection were reported.

**TABLE 4:** Frequency of adverse events

*n* (%)	Cycle 1 (*N* = 234)	Cycle 2 (*N* = 194)	Cycle 3 (*N* = 161)	All treatments (*N* = 589)
Any adverse event	15 (6.4)	8 (4.1)	3 (1.9)	26 (4.4)
Muscle weakness	6 (2.6)	5 (2.6)	3 (1.9)	14 (2.4)
Injection pain	8 (3.4)	‐	‐	8 (1.4)
Dysphagia	1 (0.4)	1 (0.5)	‐	2 (0.3)
Neck pain	‐	1 (0.5)	‐	1 (0.2)
Upper respiratory infection	‐	1 (0.5)	‐	1 (0.2)

## Discussion

The PrevU program provided physicians with the opportunity to evaluate DAXI for the treatment of CD in a real‐world setting, and to build upon evidence from the pivotal trial and open‐label safety studies^22^, ^23^ in which patient selection and treatment parameters were prescribed. The program continued for over a year to allow for optimization of treatment (including dose and inter‐injection interval) over several cycles. The experience of clinicians participating in the PrevU program confirmed the dosing, long duration of symptom relief, and the safety profile of DAXI as assessed in the phase 3 ASPEN program, but in a broader selection of patients and outside the constraints of a clinical trial.

As expected, most patients (88%) selected for treatment with DAXI were being switched from a previous BoNT (Table 1). Of these, most (76%) were considered by the treating physician to have a history of symptom breakthrough within 12 weeks of treatment with their prior BoNT. Recurrence of symptoms before the next treatment can be administered is commonly reported in the literature, and may represent up to 88% of CD patients.^12^ The percentage of patients selected for treatment in this program who experienced this “rollercoaster” effect is consistent with that previously reported.

Across the three treatment cycles, the mean re‐treatment interval was approximately 16 weeks across all cycles. Importantly, this 16‐week re‐treatment interval was observed even among patients with a history of symptom breakthrough at less than 12 weeks on their prior BoNT therapy.

Over the course of the program, physicians gradually titrated the doses of DAXI from a median starting dose of 250 U to a median dose of 300 U by Cycle 3. Interestingly, this parallels the phase 3 open‐label safety study (ASPEN‐OLS), in where 69% of patients were initiated on a dose of 250 U and 46% were titrated to a dose of 300 U.^20^

The most common strategy for switching BoNT‐experienced patients to DAXI was to use a ratio of approximately 1:1 of DAXI units to prior BoNT units (in onabotulinumtoxinA equivalents). Given that 76% of treatment‐experienced patients had symptom breakthrough before 12 weeks with a previous BoNT, it is noteworthy that in this cohort, patients and physicians settled on an average time of 16 weeks between treatments with DAXI, thus allowing for extended symptom control. As doses were optimized over three treatment cycles, the final ratio of DAXI to BoNT was approximately 1.4:1. Note that 100 units of DAXI contains approximately 0.46 ng of core neurotxin whereas 100 units of onabotulinumtoxinA contains approximately 0.73 ng of core neurotoxin. Therefore, even at a unit dose ratio of 1.4:1, DAXI would still be delivering a smaller amount of neurotoxin compared to onabotulinumtoxinA (1.4 × 0.46 = 0.64 ng core neurotoxin). The BoNT‐naïve patients were typically started at a lower initial dose, as is common in clinical practice.

Consistent with the findings from the clinical development program,^20^, ^23^ adverse event rates remained low across treatment cycles. Rates of muscle weakness (2.4%) and dysphagia (0.3%), 2 adverse events of significant concern associated with BoNT treatment of CD,^24^, ^25^, ^26^, ^27^ remained constant across DAXI treatment cycles, even as doses were increased (Table 4). The observed rates of muscle weakness and dysphagia also compare favorably to those reported in a recent real‐world, claims‐based analysis of CD patients receiving other BoNTs.^27^

The low rate of adverse events with DAXI may be related to the inclusion of the RTP004 peptide in its formulation. The peptide has a strong net positive charge and has been shown to (1) bind electrostatically to the core neurotoxin, (2) enhance binding of the toxin to the neuronal surface, and (3) increase SNAP‐25 cleavage in a dose‐dependent manner, allowing for the use of lower amounts of core neurotoxin.^8^, ^15^, ^17^, ^18^, ^28^, ^29^ Because 100 units of DAXI contains less core neurotoxin than an equivalent dose of onabotulinumtoxinA (0.46 ng vs. 0.73 ng, respectively), this lower exposure may account for the low adverse event rate and may help reduce the incidence of neutralizing antibody formation when combined with the observed 16‐week re‐injection interval. The positive charge on the peptide may also help retain the toxin in the negatively charged milieu of the injected muscle, potentially reducing diffusion to off‐target tissues.^17^

In this early experience program, for more than half of DAXI re‐treatments, physicians reported re‐treating patients to “maintain symptom relief” (Table 3), rather than waiting to “address a decline in symptom relief” or re‐emergence of symptoms. When maintaining relief, physicians generally titrated DAXI doses more modestly, if at all (mean increase 14 U, median 0 U), in contrast to treating to relieve symptoms which had re‐emerged (mean increase 46 U, median 50 U). The achievement of a maintenance dose, at an interval of 16 weeks, in more than half of treated patients demonstrates the effectiveness of DAXI in addressing the symptom re‐emergence frequently reported with conventional BoNTs. DAXI treatment allows physicians to develop individualized treatment algorithms for patients to help achieve consistent symptom control through and beyond the 12‐week minimum dosing interval.

There remain several limitations to this real‐world analysis. Since this was not designed as a clinical study with hypotheses to be tested, validated efficacy measures, such as the Toronto Western Spasmodic Torticollis Rating Scale (TWSTRS), were not used for assessment. In addition, only total administered doses, as provided by HCPs, were summarized, while specific doses per muscle were not evaluated. Due to individual variation in the dates of DAXI initiation and length of re‐treatment intervals, the amount of information was not constant across treatment cycles. However, results of the constant cohort analysis in all patients who received three treatments closely mirrored those of the observed analyzed cases, helping to rule out bias due to selection effects. Furthermore, adverse events were reported as in usual clinical practice by the practitioner, rather than following the prescribed and systematic collection performed in pivotal trials. Therefore, adverse events may have been under‐reported relative to clinical development studies. Nevertheless, this early experience program likely included many difficult‐to‐treat patients, exemplified by the large percentage of patients with breakthrough symptoms on prior BoNT therapy. As such, this analysis provides valuable insights into dosing recommendations for both initiating and titrating therapy as well as preferred re‐treatment intervals.

Overall, the extended duration of action and safety profile observed in the DAXI phase 3 trials were reflected in the real‐world setting. The availability of DAXI may provide an option for CD patients to experience more continuous symptom relief, even in those patients with early breakthrough symptoms on other BoNT therapies.

## Author Roles

(1) Research project: 1A. Conception, 1B. Organization, 1C. Execution; (2) Statistical Analysis: 2A. Design, 2B. Execution, 2C. Review and Critique; (3) Manuscript Preparation: 3A. Writing of the first draft, 3B. Review and Critique;

L.B.: 1C, 3B.

A.E.: 1C, 3B.

R.A.H.: 1C, 3B.

H.S.L.: 1C, 3B.

P.M.: 1C, 3B.

A.T.P.: 1C, 3B.

R.K: 1A, 1B, 2A, 2C, 3A, 3B.

T.M.G.: 2A, 2B, 3B.

J.S.: 3B.

C.J.G.: 1A, 3B.

D.A.H.: 1A, 3B.

## Disclosures

**Ethical Compliance Statement:** The collection of information in this study adhered to the principles of the Declaration of Helsinki. As this program was based on physician experience during real‐world clinical practice, there was no study protocol and approval by an ethics review board was not required. As all data were obtained as part of patients’ routine medical care, informed patient consent was not necessary for this work. We confirm that we have read the Journal's position on issues involved in ethical publication and affirm that this work is consistent with those guidelines.

**Funding Sources and Conflict of Interest:** The authors did not receive honoraria or payments for authorship. This analysis was funded by Revance Therapeutics, Inc., Nashville, TN, who were involved in the study design, data analysis, writing, and the decision to submit the article for publication. T.M.G., J.S., C.J.G., R.K., and D.A.H. are employees or former employees of Revance. Writing and editorial assistance was provided to the authors by Serina Stretton, PhD, CMPP, and Rania Kairouz‐Wahbe, PhD, of Envision Pharma Group and was funded by Revance, Nashville, TN, USA.

**Financial Disclosures for the Previous 12 Months:** A.T.P. received research grants and served as a consultant for AbbVie, Ipsen, and Revance Therapeutics, Inc.; and served as a speaker for AbbVie and Ipsen. P.M. received research support and consulting fees from AbbVie, Ipsen, Merz, and Revance Therapeutics, Inc.; and served on the speaker's bureau for AbbVie. A.E. received consulting and speaker honoraria from AbbVie, Acorda, Arbor, Biohaven, Biovie, Ipsen, Kyowa Kirin, Mitsubishi Tanabe Pharma America, Supernus, Teva, US World Meds, and XW Labs. R.A.H. received speaking fees from AbbVie, Amneal Pharmaceuticals, Cerevel, Kyowa Kirin, Neurocrine Biosciences, and Supernus; received consulting fees from AbbVie, AgeX Therapeutics, Amneal Pharmaceuticals, Avanex, Biogen, BlueRock Therapeutics, Canfield Scientific, Severance, Cerevel, Clario, Forsee Pharmaceuticals, HanAll Biopharma, Inhibikase, Intrance (PSG), Jazz Pharmaceuticals, KeifeRx, Kyowa Kirin, MDCE Suzhou, MedRhythms, Merz, Mitsubishi Tanabe Pharma, Nano PharmaSolutions, Neurocrine Biosciences, Neuroderm, NDP pharmaceuticals, Pharma Two B, PhotoPharmics, Regenxbio, Revance Therapeutics, Inc., Serina Therapeutics, Stoparkinson, Supernus Pharmaceuticals, Theravance, Tolmar, Tremor Research Group, Tris Pharma, TrueBinding, UCB Pharma, Vivifi Medical, and Zambon; serves on a scientific advisory board for Inhibikase, PhotoPharmics, and Stoparkinson; held stocks in Revance Therapeutics, Inc. and has stock options in Axial Therapeutics, Enterin, and Inhibikase; has received intellectual property interests from a PD Diary through his university; and acknowledges a Center of Excellence grant from the Parkinson Foundation; R.A.H.'s university has received research support from AbbVie, EON Biopharma, Alexza Pharmaceuticals, Annovis Bio, Biogen, Biogen MA, Bukwang Pharmaceutical, Cavion, Cerevance, Cerevel Therapeutics, Enterin, F. Hoffmann‐La Roche, Genentech, Global Kinetics Corporation, Inhibikase, Michael J. Fox Foundation for Parkinson's Research, National Parkinson's Foundation, Neuraly, Neuroderm, Sage Therapeutics, Sanofi Pharmaceuticals, Scion NeuroStim, UCB, and UCB Pharma. H.S.L. has served as a consultant for Revance Therapeutics, Inc and has received research support from Boston Scientific and Rune Labs. L.B. has received consulting and speaker fees from AbbVie, Acadia Healthcare, Acorda Therapeutics, Amneal Pharmaceuticals, Ipsen, Kyowa Kirin, Merz, Neurocrine Biosciences, Revance Therapeutics, Supernus Pharmaceuticals, and Teva. T.M.G., J.S., C.J.G., R.K., and D.A.H. are employees or former employees of Revance Therapeutics, Inc.

## Data Availability

Anonymized data not provided in this manuscript may be shared at the request of any qualified investigator.
